# MED: a new non-supervised gene prediction algorithm for bacterial and archaeal genomes

**DOI:** 10.1186/1471-2105-8-97

**Published:** 2007-03-16

**Authors:** Huaiqiu Zhu, Gang-Qing Hu, Yi-Fan Yang, Jin Wang, Zhen-Su She

**Affiliations:** 1 State Key Lab for Turbulence and Complex Systems and Department of Biomedical Engineering, Peking University, Beijing 100871, China; 2 Center for Theoretical Biology and Department of Physics, Peking University, Beijing 100871, China; 3 State Key Lab of Pharmaceutical Biotechnology, Nanjing University, Nanjing 210093, China; 4 Department of Mathematics, UCLA, Los Angeles, CA 90095, USA

## Abstract

**Background:**

Despite a remarkable success in the computational prediction of genes in Bacteria and Archaea, a lack of comprehensive understanding of prokaryotic gene structures prevents from further elucidation of differences among genomes. It continues to be interesting to develop new *ab initio *algorithms which not only accurately predict genes, but also facilitate comparative studies of prokaryotic genomes.

**Results:**

This paper describes a new prokaryotic genefinding algorithm based on a comprehensive statistical model of protein coding Open Reading Frames (ORFs) and Translation Initiation Sites (TISs). The former is based on a linguistic "Entropy Density Profile" (EDP) model of coding DNA sequence and the latter comprises several relevant features related to the translation initiation. They are combined to form a so-called Multivariate Entropy Distance (MED) algorithm, MED 2.0, that incorporates several strategies in the iterative program. The iterations enable us to develop a non-supervised learning process and to obtain a set of genome-specific parameters for the gene structure, before making the prediction of genes.

**Conclusion:**

Results of extensive tests show that MED 2.0 achieves a competitive high performance in the gene prediction for both 5' and 3' end matches, compared to the current best prokaryotic gene finders. The advantage of the MED 2.0 is particularly evident for GC-rich genomes and archaeal genomes. Furthermore, the genome-specific parameters given by MED 2.0 match with the current understanding of prokaryotic genomes and may serve as tools for comparative genomic studies. In particular, MED 2.0 is shown to reveal divergent translation initiation mechanisms in archaeal genomes while making a more accurate prediction of TISs compared to the existing gene finders and the current GenBank annotation.

## Background

At the time of this writing nearly 400 complete prokaryotic genomes, including 28 archaeal ones, have been deposited in the GenBank database. Driven by the acceleration in genome sequencing, several successful gene prediction programs have been designed for prokaryotic genomes, such as the GeneMark series [1,2], Glimmer [3,4] and ZCURVE [5]. The first two algorithms employ an inhomogeneous Markov model for short DNA segments (*i.e. k*-tuples), from which an estimate of the likelihood for the segment to belong to a protein coding sequence is derived after a training with existing gene data. ZCURVE is based on a Z-curve representation of a segment of DNA sequence, which is a specific statistic of the whole sequence. This category of methods are capable of an *ab initio *prediction of genes for newly sequenced genomes, and they integrate the information from sequence statistics with signal identification [6-8]. Another broad category of gene prediction methods developed earlier are based on similarity search, which comprises such programs as BLASTX and FASTA [9,10]. ORPHEUS [11] is a typical system that utilizes similarity-based algorithms. Larsen and Krogh [12] have developed an EasyGene system that combines the two approaches, using the classical Hidden Markov Model (HMM) in combination with protein similarity search BLASTP. However, not all of genes (70–80%) in newly sequenced prokaryotic genomes show significant similarity with function-known genes, it is still a practical goal to develop the *ab initio *gene-finding algorithms that require no prior knowledge of the genes of the prokaryotic genome of interest [8].

Despite the success of the existing prokaryotic gene finders, systematic bias is significantly due to the necessity of pre-training the prediction program with existing gene data [13]. This is particularly serious for gene starts and short genes whose computational annotation is still quite suspicious [14,15]. The systematic bias is also notable for GC-rich genomes to be annotated, since the sequence patterns of GC-rich genomes appear to be different to those with lower GC content [5]. It is argued that the number of potential errors in the annotation may be much higher than what is usually believed [7]. A serious case is the archaeal genome *Aeropyrum pernix*. Initially, all ORFs longer than 300 bps were annotated as coding genes by the original authors who submitted the data to GenBank, but significant disagreements arose later from several computational prediction groups [16]. Moreover, both recent experiments and *in silico *analyses have shown that the genomic patterns and hence the mechanisms of translation initiation process are proved to be diversified in Archaea [17,18]. Thus the prediction of archaeal genes is far from being a solved problem. It is commonly believed that a better understanding of the structures of genes both in bacterial and archaeal genomes with a wider variety of GC content is an essential component for improvements of gene prediction.

In this paper, we present a new non-supervised gene prediction algorithm for bacterial and archaeal genomes. The algorithm aims to develop a comprehensive statistical model with a clear picture of the architecture of prokaryotic genes, in which the biological understanding is explicitly presented. It is based on the model of protein coding Open Reading Frames (ORFs) and Translation Initiation Sites (TISs). The former is based on a linguistic "Entropy Density Profile" (EDP) model [19] of coding DNA sequence and the latter comprises several relevant features related to the initiation of a translation process. They are combined to form a so-called Multivariate Entropy Distance (MED) algorithm, MED 2.0, that incorporates several strategies in the iterative program. The iterations enable an non-supervised learning process and to obtain a set of genome-specific parameters before making the prediction of genes. The main advantage of the algorithm is that it gives efficient and accurate prediction of genes particularly for genomic GC-rich species and Archaea, and of gene-related properties such as the divergent translational initiation signals and genome-specific usage of start codon ATG, GTG and TTG without any training data. This feature of our work, namely a close link between the gene prediction method and the biological understanding of gene structure, is believed to be helpful in understanding the genomic comparison and evolution for Bacteria and Archaea.

The model developed here includes an EDP model for the coding potential of an ORF, and a TIS model for the gene starts. We begin by briefly reviewing EDP model used in our previous work [19]. We then emphasize on describing the TIS model, and the implementation of the gene prediction system MED 2.0 based on an iterative learning algorithm.

### The EDP model for coding potential of ORF

The EDP model is a global statistical description for a DNA sequence, which employs a Shannon's artificial linguistic description for a DNA sequence of finite length like an ORF. It is different from the Markov model-based local approach that usually selects a few local features of the class of sequences. Instead of the amino acid composition {*p*_*i*_} (*i *= 1, ..., 20) for an ORF, an EDP vector *S *= {*s*_*i*_} inferred from {*p*_*i*_} is used to represent the sequence with an emphasis on the information content, where *i *is the index of the twenty amino acids. The EDP {*s*_*i*_} is defined by [19].

si=−1Hpilog⁡pi,     (1)
 MathType@MTEF@5@5@+=feaafiart1ev1aaatCvAUfKttLearuWrP9MDH5MBPbIqV92AaeXatLxBI9gBaebbnrfifHhDYfgasaacH8akY=wiFfYdH8Gipec8Eeeu0xXdbba9frFj0=OqFfea0dXdd9vqai=hGuQ8kuc9pgc9s8qqaq=dirpe0xb9q8qiLsFr0=vr0=vr0dc8meaabaqaciaacaGaaeqabaqabeGadaaakeaacqWGZbWCdaWgaaWcbaGaemyAaKgabeaakiabg2da9iabgkHiTmaalaaabaGaeGymaedabaGaemisaGeaaiabdchaWnaaBaaaleaacqWGPbqAaeqaaOGagiiBaWMaei4Ba8Maei4zaCMaemiCaa3aaSbaaSqaaiabdMgaPbqabaGccqGGSaalcaWLjaGaaCzcamaabmaabaGaeGymaedacaGLOaGaayzkaaaaaa@4267@

where H=−∑j=120pjlog⁡pj
 MathType@MTEF@5@5@+=feaafiart1ev1aaatCvAUfKttLearuWrP9MDH5MBPbIqV92AaeXatLxBI9gBaebbnrfifHhDYfgasaacH8akY=wiFfYdH8Gipec8Eeeu0xXdbba9frFj0=OqFfea0dXdd9vqai=hGuQ8kuc9pgc9s8qqaq=dirpe0xb9q8qiLsFr0=vr0=vr0dc8meaabaqaciaacaGaaeqabaqabeGadaaakeaacqWGibascqGH9aqpcqGHsisldaaeWaqaaiabdchaWnaaBaaaleaacqWGQbGAaeqaaaqaaiabdQgaQjabg2da9iabigdaXaqaaiabikdaYiabicdaWaqdcqGHris5aOGagiiBaWMaei4Ba8Maei4zaCMaemiCaa3aaSbaaSqaaiabdQgaQbqabaaaaa@40EE@ is the Shannon entropy, and *j *= 1, ..., 20 also represents the index of the twenty amino acids.

For a set of sequences such as all coding and noncoding ORFs from a given genome, the vectors {*s*_*i*_} scatter widely in the 20-dimensional EDP phase space. Our basic working hypothesis is that the EDP vectors for coding ORFs form separate clusters from those for noncoding ORFs, for a possible reason that they moved along separate paths during the evolution due to different selection pressure. If this hypothesis is true, one can then develop a clustering method in the EDP phase space that discriminates the coding ORFs from the noncoding ones. The success of the method confirms the validity of this hypothesis. Our previous study shows that there exists indeed one universal center representative of coding ORFs and one universal center of noncoding ORFs in the EDP phase space for dozens of analysed species [19]. For GC-rich genomes, our current research based on Principal Component Analysis (PGA) shows that ORFs form six clusters in the EDP phase space due to the genomic GC content bias (data not shown), one for coding ORFs while other five for non-coding ORFs, which is also consistent with a previous study [20]. Thus the noncoding ORFs of GC-rich genomes are represented with five universal centers in the EDP phase space. Following the ZCURVE algorithm [5], we refer to the GC-rich genome as a genome with GC content higher than 56%. Consequently, the universal centers allow us to construct an iterative procedure to define a refined coding potential [19]. This procedure begins by finding a set of "root" coding and noncoding ORFs ({*S*(*k*)^*coding*^} and {*S*(*l*)^*noncoding*^}), which have been demonstrated with high reliability based on the universal EDP centers [19], and then builds a coding potential of a DNA sequence *S *like an ORF as the ratio of the distance to each of the EDP clusters

ΦEDP(S)=min⁡kD(S,S(k)coding)min⁡lD(S,S(l)noncoding),     (2)
 MathType@MTEF@5@5@+=feaafiart1ev1aaatCvAUfKttLearuWrP9MDH5MBPbIqV92AaeXatLxBI9gBaebbnrfifHhDYfgasaacH8akY=wiFfYdH8Gipec8Eeeu0xXdbba9frFj0=OqFfea0dXdd9vqai=hGuQ8kuc9pgc9s8qqaq=dirpe0xb9q8qiLsFr0=vr0=vr0dc8meaabaqaciaacaGaaeqabaqabeGadaaakeaacqqHMoGrdaahaaWcbeqaaiabdweafjabdseaejabdcfaqbaakiabcIcaOiabdofatjabcMcaPiabg2da9maalaaabaGagiyBa0MaeiyAaKMaeiOBa42aaSbaaSqaaiabdUgaRbqabaGccqWGebardaqadaqaaiabdofatjabcYcaSiabdofatjabcIcaOiabdUgaRjabcMcaPmaaCaaaleqabaGaem4yamMaem4Ba8MaemizaqMaemyAaKMaemOBa4Maem4zaCgaaaGccaGLOaGaayzkaaaabaGagiyBa0MaeiyAaKMaeiOBa42aaSbaaSqaaiabdYgaSbqabaGccqWGebardaqadaqaaiabdofatjabcYcaSiabdofatjabcIcaOiabdYgaSjabcMcaPmaaCaaaleqabaGaemOBa4Maem4Ba8MaemOBa4Maem4yamMaem4Ba8MaemizaqMaemyAaKMaemOBa4Maem4zaCgaaaGccaGLOaGaayzkaaaaaiabcYcaSiaaxMaacaWLjaWaaeWaaeaacqaIYaGmaiaawIcacaGLPaaaaaa@6C5F@

where *k *and *l *are the indices of two clusters of root coding and noncoding ORFs, *D*(*S, S'*) can be defined by the Euclidean distance between two EDP vectors *S *= {*s*_*i*_} and *S' *= {s′i
 MathType@MTEF@5@5@+=feaafiart1ev1aaatCvAUfKttLearuWrP9MDH5MBPbIqV92AaeXatLxBI9gBaebbnrfifHhDYfgasaacH8akY=wiFfYdH8Gipec8Eeeu0xXdbba9frFj0=OqFfea0dXdd9vqai=hGuQ8kuc9pgc9s8qqaq=dirpe0xb9q8qiLsFr0=vr0=vr0dc8meaabaqaciaacaGaaeqabaqabeGadaaakeaacuWGZbWCgaqbamaaBaaaleaacqWGPbqAaeqaaaaa@2FAE@} as

*D*(*S*, *S'*) = (∑_*i *_(*s*_*i *_- s′i
 MathType@MTEF@5@5@+=feaafiart1ev1aaatCvAUfKttLearuWrP9MDH5MBPbIqV92AaeXatLxBI9gBaebbnrfifHhDYfgasaacH8akY=wiFfYdH8Gipec8Eeeu0xXdbba9frFj0=OqFfea0dXdd9vqai=hGuQ8kuc9pgc9s8qqaq=dirpe0xb9q8qiLsFr0=vr0=vr0dc8meaabaqaciaacaGaaeqabaqabeGadaaakeaacuWGZbWCgaqbamaaBaaaleaacqWGPbqAaeqaaaaa@2FAE@)^2^)^1/2^, *i *= 1, ..., 20.

A noteworthy feature of this coding potential is that it is able to generate a cluster with non-trivial boundary and thus achieve a classification of the two classes of sequences with remarkable accuracy [19]. For this reason, the EDP model has been successfully used to produce training ORF set in the newest version of Glimmer method [21].

### The TIS model for translation start site of prokaryotic genes

The TIS model in MED 2.0, which is a further extension of the RBS model described earlier [22], makes a conscious effort to model the following prominent features for prokaryotic gene structure: (1) Upstream to the TIS there exists one or a few common motifs that act as the binding sites for the initiation of translation or transcription, these common motifs usually have a position-specific distribution in upstream region from the start sites; (2) Among various genomes, the usage of start codon ATG, GTG and TTG is different; (3) For ORFs with multiple candidate start codons, the leftmost start codons are usually the TISs. However, those start codons located after the leftmost ones are also chosen to be TISs according to different probability; (4) The (overlapping) distance between the neighbor genes has a characteristic probability distribution; (5) For GC-rich genomes, there exists a high GC content at the codon first and third position due to the GC content bias [4,5,8,11,12,22,23]. These features are included in the TIS model, allowing us to iteratively find the true TIS after the determination of the right 3' end coding ORFs. It is notable that no training data is required to fix the model parameters. The various probabilities mentioned above are obtained by non-supervised learning during the iteration. It is intriguing to note also that these probabilities are genome-specific quantities and their differentiation between genomes may likely be interesting and significant for comparative genomic studies.

For the RBS model in our previous work [22], two likelihood functions are defined, one being associated with the Shine-Dalgarno (SD) motif [24], Φ^*motif*^, and another being directly related to the start codon, Φ^*start*^. The total likelihood reads Φ^*total *^= Φ^*motif *^+ Φ^*start*^. We have made several crucial extensions of that model as follows.

First, many Archaea (*e.g. S. solfataricus*) are detected to have both SD signals of bacterial type in near upstream region and box A motifs of eukaryotic type in the further upstream region from TIS. So, MED 2.0 defines two motif searching regions, *V*^*up *^= [-35, -15] and *V*^*low *^= [-20, -1], and carries out search for candidate motifs separately in each region.

Secondly, a new likelihood function is defined to describe the probability that two ORFs overlap, which is given by a distribution of the distance between a start codon and the STOP codon of the immediate upstream ORF.

Thirdly, for GC-rich genomes we use a parameter *CP *defined by formula (3) to describe the difference between the coding region *V*^*c *^= [1, 90] downstream and the noncoding region *V*^*nc *^= [-90, -1] upstream to TIS. It has long been known that the sequence patterns of genomes with GC-rich content show differences to those with normal or low GC content. As a result, high GC content at the codon third and first position has been reported for GC-rich genomes [5,8]. Similar to Nishi *et al*. [8], we refer to *GC*(*i*) as the G+C occurrence at the *i*th codon position, where *i *= 1, 2 or 3, then parameter θ=GC(1)+GC(3)GC(1)+GC(2)+GC(3)
 MathType@MTEF@5@5@+=feaafiart1ev1aaatCvAUfKttLearuWrP9MDH5MBPbIqV92AaeXatLxBI9gBaebbnrfifHhDYfgasaacH8akY=wiFfYdH8Gipec8Eeeu0xXdbba9frFj0=OqFfea0dXdd9vqai=hGuQ8kuc9pgc9s8qqaq=dirpe0xb9q8qiLsFr0=vr0=vr0dc8meaabaqaciaacaGaaeqabaqabeGadaaakeaaiiGacqWF4oqCcqGH9aqpdaWcaaqaaiabdEeahjabdoeadjabcIcaOiabigdaXiabcMcaPiabgUcaRiabdEeahjabdoeadjabcIcaOiabiodaZiabcMcaPaqaaiabdEeahjabdoeadjabcIcaOiabigdaXiabcMcaPiabgUcaRiabdEeahjabdoeadjabcIcaOiabikdaYiabcMcaPiabgUcaRiabdEeahjabdoeadjabcIcaOiabiodaZiabcMcaPaaaaaa@4A17@ is used to describe the nucleotide usage at the codon first and third position. For both two regions *V*^*c *^and *V*^*nc*^, we treat them as a set of codon sequences regardless of the fact that the *V*^*nc *^are noncoding sequences. Our analysis shows that the probability distribution of usage *P*_*c*_(*θ*) for coding region *V*^*c *^is significantly different from that of usage *P*_*nc*_(*θ*) of noncoding region *V*^*nc*^. Thus for a DNA sequence with a usage *θ*, the *CP*(*θ*) parameter is designed to describe the probability of the sequence belonging to coding region as follow

*CP*(*θ*) = *P*_*c*_(*θ*)/(*P*_*c*_(*θ*) + *P*_*nc*_(*θ*)),     (3)

where 0 ≤ *CP*(*θ*) ≤ 1. To evaluate the likelihood of a start codon as TIS, we calculate the nucleotide usage *θ*_*c *_and *θ*_*nc *_for its downstream region [1, 90] and upstream region [-90, -1] separately. Thus the likelihood function is defined by the formula

ΦGCstart
 MathType@MTEF@5@5@+=feaafiart1ev1aaatCvAUfKttLearuWrP9MDH5MBPbIqV92AaeXatLxBI9gBaebbnrfifHhDYfgasaacH8akY=wiFfYdH8Gipec8Eeeu0xXdbba9frFj0=OqFfea0dXdd9vqai=hGuQ8kuc9pgc9s8qqaq=dirpe0xb9q8qiLsFr0=vr0=vr0dc8meaabaqaciaacaGaaeqabaqabeGadaaakeaacqqHMoGrdaqhaaWcbaGaem4raCKaem4qameabaGaem4CamNaemiDaqNaemyyaeMaemOCaiNaemiDaqhaaaaa@3782@ = [1 - *CP*(*θ*_*nc*_)] * *CP*(*θ*_*n*_),     (4)

where 0 ≤ ΦGCstart
 MathType@MTEF@5@5@+=feaafiart1ev1aaatCvAUfKttLearuWrP9MDH5MBPbIqV92AaeXatLxBI9gBaebbnrfifHhDYfgasaacH8akY=wiFfYdH8Gipec8Eeeu0xXdbba9frFj0=OqFfea0dXdd9vqai=hGuQ8kuc9pgc9s8qqaq=dirpe0xb9q8qiLsFr0=vr0=vr0dc8meaabaqaciaacaGaaeqabaqabeGadaaakeaacqqHMoGrdaqhaaWcbaGaem4raCKaem4qameabaGaem4CamNaemiDaqNaemyyaeMaemOCaiNaemiDaqhaaaaa@3782@ ≤ 1. Note that the score ΦGCstart
 MathType@MTEF@5@5@+=feaafiart1ev1aaatCvAUfKttLearuWrP9MDH5MBPbIqV92AaeXatLxBI9gBaebbnrfifHhDYfgasaacH8akY=wiFfYdH8Gipec8Eeeu0xXdbba9frFj0=OqFfea0dXdd9vqai=hGuQ8kuc9pgc9s8qqaq=dirpe0xb9q8qiLsFr0=vr0=vr0dc8meaabaqaciaacaGaaeqabaqabeGadaaakeaacqqHMoGrdaqhaaWcbaGaem4raCKaem4qameabaGaem4CamNaemiDaqNaemyyaeMaemOCaiNaemiDaqhaaaaa@3782@ is calculated only for the genomes with GC content higher than 56%, and this item is added into the start codon likelihood function Φ^*start *^developed in our previous work [22].

Fourthly, MED 2.0 introduces a measure of signal strength in order to counter-balance the genomic nucleotide content bias. Let *T *be the ensemble of all *l*-mers in the set *V *of upstream sequences of all candidate coding ORFs, and for each *l*-mer *t *∈ *T*, denote by p^
 MathType@MTEF@5@5@+=feaafiart1ev1aaatCvAUfKttLearuWrP9MDH5MBPbIqV92AaeXatLxBI9gBaebbnrfifHhDYfgasaacH8akY=wiFfYdH8Gipec8Eeeu0xXdbba9frFj0=OqFfea0dXdd9vqai=hGuQ8kuc9pgc9s8qqaq=dirpe0xb9q8qiLsFr0=vr0=vr0dc8meaabaqaciaacaGaaeqabaqabeGadaaakeaacuWGWbaCgaqcaaaa@2E25@(*t*) the observed frequency in the region *V*. Denote also by p¯
 MathType@MTEF@5@5@+=feaafiart1ev1aaatCvAUfKttLearuWrP9MDH5MBPbIqV92AaeXatLxBI9gBaebbnrfifHhDYfgasaacH8akY=wiFfYdH8Gipec8Eeeu0xXdbba9frFj0=OqFfea0dXdd9vqai=hGuQ8kuc9pgc9s8qqaq=dirpe0xb9q8qiLsFr0=vr0=vr0dc8meaabaqaciaacaGaaeqabaqabeGadaaakeaacuWGWbaCgaqeaaaa@2E2D@(*t*) the frequency of the same *l*-mer if it is a random combination of its nucleotides; thus, p¯
 MathType@MTEF@5@5@+=feaafiart1ev1aaatCvAUfKttLearuWrP9MDH5MBPbIqV92AaeXatLxBI9gBaebbnrfifHhDYfgasaacH8akY=wiFfYdH8Gipec8Eeeu0xXdbba9frFj0=OqFfea0dXdd9vqai=hGuQ8kuc9pgc9s8qqaq=dirpe0xb9q8qiLsFr0=vr0=vr0dc8meaabaqaciaacaGaaeqabaqabeGadaaakeaacuWGWbaCgaqeaaaa@2E2D@(*t*) can be estimated from single nucleotide composition measured from the whole genome. Similarly to Fuglsang [25], we refer to *X*(*t*) = (p^
 MathType@MTEF@5@5@+=feaafiart1ev1aaatCvAUfKttLearuWrP9MDH5MBPbIqV92AaeXatLxBI9gBaebbnrfifHhDYfgasaacH8akY=wiFfYdH8Gipec8Eeeu0xXdbba9frFj0=OqFfea0dXdd9vqai=hGuQ8kuc9pgc9s8qqaq=dirpe0xb9q8qiLsFr0=vr0=vr0dc8meaabaqaciaacaGaaeqabaqabeGadaaakeaacuWGWbaCgaqcaaaa@2E25@(*t*) - p¯
 MathType@MTEF@5@5@+=feaafiart1ev1aaatCvAUfKttLearuWrP9MDH5MBPbIqV92AaeXatLxBI9gBaebbnrfifHhDYfgasaacH8akY=wiFfYdH8Gipec8Eeeu0xXdbba9frFj0=OqFfea0dXdd9vqai=hGuQ8kuc9pgc9s8qqaq=dirpe0xb9q8qiLsFr0=vr0=vr0dc8meaabaqaciaacaGaaeqabaqabeGadaaakeaacuWGWbaCgaqeaaaa@2E2D@(*t*))^2^/p¯
 MathType@MTEF@5@5@+=feaafiart1ev1aaatCvAUfKttLearuWrP9MDH5MBPbIqV92AaeXatLxBI9gBaebbnrfifHhDYfgasaacH8akY=wiFfYdH8Gipec8Eeeu0xXdbba9frFj0=OqFfea0dXdd9vqai=hGuQ8kuc9pgc9s8qqaq=dirpe0xb9q8qiLsFr0=vr0=vr0dc8meaabaqaciaacaGaaeqabaqabeGadaaakeaacuWGWbaCgaqeaaaa@2E2D@(*t*) as a measure of the over-representation of *t *∈ *T*. When summing over the ensemble *T*, we have a measure, *χ*^2^(*T*) for deciding whether *T*^*up*^or *T*^*low *^is more likely to contain a strong signal in *V*^*up *^or *V*^*low*^, where *T*^*up *^(or *T*^*low*^) means the ensemble of *l*-mers in the region *V*^*up *^(or *V*^*low*^). Such measure is also used to refer to a weight for each region, as *m*^*up *^= *χ*^2^(*T*^*up*^)/(*χ*^2^(*T*^*up*^) + *χ*^2^(*T*^*low*^)), and *m*^*low *^= *χ*^2^(*T*^*low*^)/(*χ*^2^(*T*^*up*^) + *χ*^2^(*T*^*low*^)) which allows us to obtain a combined measure for a given candidate start codon

Φ^*TIS *^= *m*^*up*^Φ^*up *^+ *m*^*low*^Φ^*low *^+ *m*^*start*^Φ^*start*^,     (5)

here, *m*^*start *^= *Max*(*m*^*up*^*, m*^*low*^). In above formula, score Φ^*up *^reflects the likelihood of a motif associated with transcription initiation signal in region *V*^*up *^= [-35, -15], while Φ^*low *^means the likelihood of a motif associated with translation initiation signal in region *V*^*low *^= [-20, -1], both two scores include a weight matrix scoring function and an occurrence probability in upstream region to TIS. For score Φ^*start*^, it includes weight matrix scoring function of sequences around TIS, the probability of start codon as TIS compared with the leftmost start codon, and the ORF overlap scoring function. If the genomic GC content is higher than 56%, the scoring function given by formula (4) will be added in. Note that the new likelihood function Φ^*TIS *^is able to describe both bacterial and archaeal genomes in a unified way.

Finally, a distinction is made between transcript unit internal (TUI) genes and transcript unit leader (TUL) genes. Following Tolstrup *et al*. [26] and Torarinsson *et al*. [18], we refer to TUI genes as those with a start codon separated from the stop codon of the nearest upstream gene by < 50 bps, and TUL genes otherwise. Our analysis in *S. solfataricus *shows that only SD-like motifs are found for TUI genes while box A motifs are usually found for TUL genes. In MED 2.0, each candidate start codon then definitely belongs to one of the two above categories, each of which conducts the search for motifs separately. Furthermore, MED 2.0 forms a measure

λ=(χ2(TTUIup)−χ2(TTUIlow))(χ2(TTULup)−χ2(TTULlow)),     (6)
 MathType@MTEF@5@5@+=feaafiart1ev1aaatCvAUfKttLearuWrP9MDH5MBPbIqV92AaeXatLxBI9gBaebbnrfifHhDYfgasaacH8akY=wiFfYdH8Gipec8Eeeu0xXdbba9frFj0=OqFfea0dXdd9vqai=hGuQ8kuc9pgc9s8qqaq=dirpe0xb9q8qiLsFr0=vr0=vr0dc8meaabaqaciaacaGaaeqabaqabeGadaaakeaaiiGacqWF7oaBcqGH9aqpcqGGOaakcqWFhpWydaahaaWcbeqaaiabikdaYaaakiabcIcaOiabdsfaunaaDaaaleaacqWGubavcqWGvbqvcqWGjbqsaeaacqWG1bqDcqWGWbaCaaGccqGGPaqkcqGHsislcqWFhpWydaahaaWcbeqaaiabikdaYaaakiabcIcaOiabdsfaunaaDaaaleaacqWGubavcqWGvbqvcqWGjbqsaeaacqWGSbaBcqWGVbWBcqWG3bWDaaGccqGGPaqkcqGGPaqkcqGGOaakcqWFhpWydaahaaWcbeqaaiabikdaYaaakiabcIcaOiabdsfaunaaDaaaleaacqWGubavcqWGvbqvcqWGmbataeaacqWG1bqDcqWGWbaCaaGccqGGPaqkcqGHsislcqWFhpWydaahaaWcbeqaaiabikdaYaaakiabcIcaOiabdsfaunaaDaaaleaacqWGubavcqWGvbqvcqWGmbataeaacqWGSbaBcqWGVbWBcqWG3bWDaaGccqGGPaqkcqGGPaqkcqGGSaalcaWLjaGaaCzcamaabmaabaGaeGOnaydacaGLOaGaayzkaaaaaa@6D64@

to decide which of the two translation initiation mechanisms is predominant in a genome: *λ *> 0 for either completely SD signals or completely box A consensus, and *λ *≤ 0 for a mixture of the two. Tests show that most of Bacteria and part of Archaea belong to the former, whereas many of Archaea belong to the latter. In the latter case, MED 2.0 uses the Z-score to form a normalized TIS score which allows to compare and select the right TIS. Therefore, our method allows to describe more than one type of initiation signals which are located at different upstream positions.

### The iterative learning algorithm

**Figure 1:**
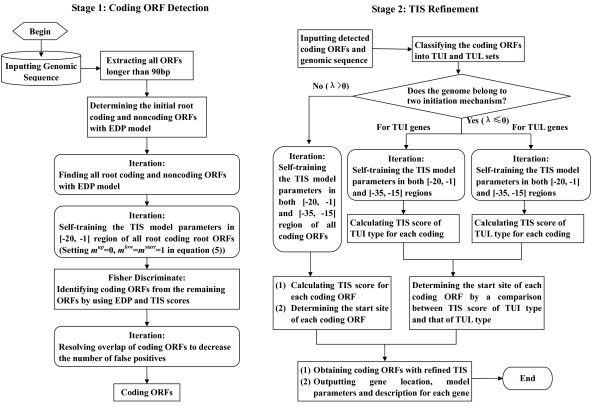
Flow chart of gene prediction process with MED 2.0 system.

The gene finding system MED 2.0 predicts genes in two stages: the coding ORF detection stage and the TIS refinement stage. The outline of sequence processing and model self-training for two stages are shown in Fig. 1. We give a summary of this algorithm as follows.

In the first step of coding ORF detection stage, all ORFs longer than 90 bps are extracted in both strands and in all three reading frames, thus to determine the EDP coding potential. As stated above, using the universal coding and noncoding EDP centers and with an iteration of consecutive root ORF finding, we decide a set of root coding/noncoding ORFs for each genome being analysed, which typically cover more than 60% of all genes with a very high reliability over 99% [19]. Note that we obtain both "seed" coding ORFs and non-coding sequences, instead of generating artificial non-coding ORFs as other methods do [5]. These seed sequences form a reliable learning set for the further analysis.

The system then goes to determine the remaining ORFs excluded from the root ORFs. We search for conservative motifs only in the near upstream region *V*^*low *^of the root coding ORFs, namely setting *m*^*up *^= 0, and *m*^*low *^= *m*^*start *^= 1 in calculating the TIS score in formula (5). The candidate start site with the highest TIS score is selected, then the parameters are recalculated using the updated start sites. This iteration process ends when no start site needs to be relocated. The parameters are then obtained and used to determine the remaining ORFs. This consists in calculating the TIS score of the remaining ORFs, and then evaluating their coding potential using a Fisher discriminant algorithm (which is trained using *B. subtilis*) in a two-dimensional plane of the EDP coding score and the TIS score. For GC-rich genomes, the Fisher discriminant algorithm is applied in a three-dimensional space of the EDP coding score, the TIS score and the *θ *score (calculated by the *θ *parameter mentioned-above), which is trained using *P. aeruginosa*. Our studies show that the discriminant coefficients are universal across prokaryotic genomes. Thus the remaining ORFs are classified into coding ORFs and noncoding ones.

To reduce the false positive in the detected coding ORFs determined from the remaining ORFs, a strategy to resolve overlap of coding ORFs is then applied. We define a quantity *ξ *to be the percentage of nucleotides overlapped with other coding ORFs in either the same or the opposite strand. The iteration goes as follows: (1) extend all the detected coding ORFs to the longest ORFs by assigning the leftmost start codon as the start of each ORF; (2) remove those ORFs with *ξ *> *ξ*^*i *^(setting *ξ*^0 ^= 99%, where *i *is the iteration step); (3) recalculate the overlapping percentage for all ORFs retained from the last step; and (4) reduce *ξ*^*i *^by 1%, then repeats from step (2). This procedure iterates until *ξ *reaches a threshold (50%, by default).

After the stage of coding ORF detection, MED 2.0 goes to the next stage of TIS refinement for all coding ORFs. The detected coding ORFs are first classified into two sets, TUI and TUL. The system considers searching for either SD motifs or box A motifs in both regions *V*^*up *^= [-35, -15] and *V*^*low *^= [-20, -1]. Then, one forms the combined TIS score in formula (5), which is calculated together with all three coefficients *m*^*up*^*, m*^*low *^and *m*^*start *^described above. For GC-rich genomes, the score Φ^*start *^includes the item of ΦGCstart
 MathType@MTEF@5@5@+=feaafiart1ev1aaatCvAUfKttLearuWrP9MDH5MBPbIqV92AaeXatLxBI9gBaebbnrfifHhDYfgasaacH8akY=wiFfYdH8Gipec8Eeeu0xXdbba9frFj0=OqFfea0dXdd9vqai=hGuQ8kuc9pgc9s8qqaq=dirpe0xb9q8qiLsFr0=vr0=vr0dc8meaabaqaciaacaGaaeqabaqabeGadaaakeaacqqHMoGrdaqhaaWcbaGaem4raCKaem4qameabaGaem4CamNaemiDaqNaemyyaeMaemOCaiNaemiDaqhaaaaa@3782@ in formula (4). Then the global genomic measure *λ *is used to decide which of the initiation mechanisms is more appropriate for the genome studied. If *λ *> 0, the self-training of TIS model parameters runs in both *V*^*up *^and *V*^*low *^for all detected coding ORFs, we select the SD-like motif as the main mechanism if *χ*^2^(*T*^*up*^) <*χ*^2^(*T*^*low*^), or the box A motif otherwise. For each coding ORF, the TIS score in formula (5) is calculated for all candidate start codons, then the start codon with the highest score is selected as the predicted TIS. If *λ *≤ 0, the self-training is run with *V*^*up *^and *V*^*low *^together, but for TUI and TUL genes independently. For each coding ORF, the normalized scores of both TUI type and TUL type are calculated, and then compared to select one with higher score as the most likely start codon to relocate the gene start.

## Results and Discussion

### Genome sequences and reliable gene datasets

The bacterial and archaeal genomes and their annotations used in this paper were downloaded from the GenBank Release 149.0 in 2006.

We have selected experimentally confirmed genes as benchmarks. For *E. coli*, we have used two datasets, the *EcoGene *[27] and the *Link *dataset [28], with their newest version (854 proteins in *EcoGene *and 195 N-terminally confirmed genes in *Link*). For *B. subtilis*, following the standard by Besemer *et al*. [23], we have taken it as one of a few annotated complete genomes that can be used to evaluate the performance of exact gene prediction. Thus we denoted all 4,100 genes annotated in GenBank as *Bsub_All *dataset. For verifying short genes, we have chosen *Bsub123, Bsub72 *and *Bsub51 *datasets, which are selected from the *B. subtilis *and verified by protein similarity search [23]. We have extracted 58 short genes from the 854 genes of *EcoGene*, denoted as *EcoGene_short*, to evaluate short gene prediction for *E. coli*.

For GC-rich genomes, we have built two data sets, *Mtub66 *and *Paer107*. The former includes 66 reliable genes with confirmed TISs in *M. tuberculosis *with GC content of 65.6%, while the latter has 107 such genes in *P. aeruginosa *with GC content of 66.6%. For archaeal genomes, we have built a data set *SolfGene *including 56 reliable genes in *S. solfataricus *with confirmed TISs. The three data sets *Mtub68, Paer111 *and *SolfGene*, as well as the process of determining these genes with their TISs confirmed by N-terminal protein sequencing or inferred from experimental evidences, can be accessed through [29].

### Other benchmark programs

In order to benchmark MED 2.0, we have tested four gene-finding methods (including five programs), Glimmer (includes the latest published version Glimmer 2.02 [4] and the newest release Glimmer 3.02 from website [21]), GeneMarkS [23], ZCURVE [5] and EasyGene [12], to make a comparison of the performance of gene prediction. The former three methods belong to the same category of our system as the *ab initio *gene prediction methods, while the EasyGene belongs to another category of methods using the extrinsic information by means of similarity search [12].

Glimmer 2.02 was downloaded from [21] and installed locally. We ran it following the instructions given in the distribution file. A post-processor RBSfinder [30] has been designed to further improve the TIS prediction by Glimmer 2.02. Thus, RBSfinder was used to process the original output of Glimmer 2.02 and the refined TISs are then taken as predicted ones by Glimmer system. RBSfinder is accessible via the website at [31]. Herein, RBSfinder was run repeatedly until over 99% of gene starts remain unchanged. Recently, the newest version of Glimmer, *i.e*. V3.02, has been released with several algorithmic changes to reduce the number of false positive predictions and to improve the accuracy of TIS predictions. Although the article describing Glimmer 3.02 has not yet been found from publication, a locally executable program is current available from [21]. In order to have a comprehensive comparison with Glimmer method, Glimmer 3.02 was further included herein as benchmark program. We downloaded the predictions by Glimmer 3.02 from its website for all genomes studied in this paper.

The software ZCURVE 1.0 is freely available at [32]. It was executed on our PC following the instruction file.

GeneMarkS provides only an online service of genomic annotation, instead of a local executable program. The newest results on several test sets mentioned above for *E. coli *and *B. subtilis *by the current GeneMarkS have been greatly improved than that of its initial version [23]. Similarly, there is still no new literature to report the improved version of GeneMarkS. However we adopted all the unpublished results returned to us via Email with the current online analysis behind the interface [33], to compare with our program.

Similar to GeneMarkS, EasyGene is only accessible via its web interface, and the pre-trained models are available for only 27 genomes [34]. However, the EasyGene web server has provided predictions for hundreds of chromosomes. Therefore, for comparison purpose, EasyGene predictions for a total of 112 genomes were downloaded from its website [34] available at the time we prepared our paper.

### Accuracy of gene detection and interpretation of genome-specific model parameters

To illustrate the prediction accuracy of MED 2.0, we first present the comparison against the GenBank annotation for the 3' end match. Two independent quantities, Sn (sensitivity) and Sp (specificity), are defined to evaluate the performance of a gene finder at gene level as:

*Sn *= *TP*/(*TP *+ *FN*), *Sp *= *TP*/(*TP *+ *FP*).     (7)

Here, *TP, FP*, and *FN *are the number of true positive, false positive, and false negative, respectively.

The MED 2.0 program has been run on all the complete bacterial and archaeal genomes currently available on GenBank. Additional File 1 includes the prediction results on forty representative genomes, comprising 28 Bacteria and 12 Archaea. Selected organisms cover most of the taxonomic groups, including Crenarchaeota, Euryarchaeota, Nanoarchaeota for Archaea and *α*/*β*/*γ*/*ε*-proteobacteria, Actinobacteria, Bacteroidetes/Chlorobi, for Bacteria [35]. For the forty species, MED 2.0 achieves an average sensitivity of 97.6% and specificity of 87.8%. The results of Glimmer 2.02, Glimmer 3.02, ZCURVE 1.0 and the newest GeneMarkS for the forty genomes as a comparison are not shown in Additional File 1, but may be found in our website. On the average, the newest version of GeneMarkS gives the same level of sensitivity (98.0%) and the highest specificity (93.0%), while Glimmer 2.02 and ZCURVE 1.0 give the sensitivity of 98.7% and 98.0%, and the specificity of 83.1% and 82.9%, respectively. As reported by EasyGene's website, the average sensitivity and specificity are 95.4% and 96.6% respectively for 29 of the 40 genomes. Compared with Glimmer 2.02 on average, Glimmer 3.02 significantly reduces the false positives (Sp = 92.3%) with almost no decreasing of the sensitivity of 97.7%. The average sensitivity and specificity of GeneMarkS reported in the original version [23], which was run on eight genomes including *E. coli, B. subtilis *and *A. fulgidus*, are 98.3% and 91.3%, respectively. A full comparison for 205 genomes has also been listed at our web page. For the 205 genomes, MED 2.0 gives the average sensitivity of 95.7% and specificity of 83.1%, while Glimmer 2.02 gives 98.0% and 79.8%, ZCURVE 1.0 gives 96.7% and 81.4%, Glimmer 3.02 gives 96.0% and 88.9%, GeneMarkS gives 96.0% and 91.3%, and finally EasyGene gives 93.5% and 96.1% averaged over 112 genomes, respectively. However, we argue that the higher specificity of the latter three programs is not unrelated to the fact that they are highly optimized with the GenBank annotation. It is clear that MED 2.0 is competitive with Glimmer 2.02 and ZCURVE 1.0, while the specificity of prediction is a little higher than both of them. Both Glimmer 3.02 and the newest GeneMarkS present rather high accuracies of sensitivity and specificity against the current GenBank annotation for the 3' end match, although there are not yet publications to report them. Using the extrinsic information of similarity search, EasyGene is able to extremely raise the specificity at the cost of its sensitivity slightly lower than the *ab initio *gene prediction methods. It should be noted that EasyGene has included many a confirmed gene of the query-genome as training set by using BLASTP to search for significant protein matches in Swiss-Prot [12], which makes an essential distinction between such a method and the *ab initio *gene prediction method such as MED 2.0. For instance, there are about 65% of all 4,329 genes in *E. coli *predicted by EasyGene have been taken as the training set. Therefore a comparison against all genes in the benchmark would over-estimate EasyGene's prediction performance. To discuss the predicting performance on the current GenBank annotation, we should point out that it can not be free from the bias since both Glimmer and GeneMark series have been widely used or involved in the GenBank annotation pipeline. The statistical analysis shows that there are over 134 genomes from GenBank before 2006 are in this case. While the comparison against the GenBank annotation has played a role during the early development of the computational prediction programs, this comparison becomes increasingly suspicious as its bias becomes evident (towards the programs which are used to create the GenBank annotation file).

Since the GenBank annotation is not fully accurate, further evaluation is performed based on the function-known genes which have more reliable annotations. For each genome, the function-known genes are selected from GenBank by excluding those with product descriptions with any of the key words as "-like", "conserved", "hypothetical", "homolog", "probable", "possible", "predicted", "putative", "similarity" and "unknown". The ratio of the number of these genes predicted correctly by MED 2.0 is also listed in Additional File 1. Note that the specificity is meaningless in this case. As we can see, the average accuracy for MED 2.0 is 99.1%, while the average accuracy for Glimmer 2.02, Glimmer 3.02, ZCURVE 1.0, EasyGene (averaged over 29 of 40 genomes) and the newest GeneMarkS is 99.4%, 99.2%, 98.9%, 98.3% and 99.3% (data not shown in Additional File 1), respectively. For 205 genomes analysed, the average accuracy for MED 2.0, Glimmer 2.02, Glimmer 3.02, ZCURVE 1.0, EasyGene and the newest GeneMarkS may be calculated, which gives 98.5%, 99.4%, 98.5%, 98.7%, 98.1% and 98.6% respectively, Clearly, they are all at the same level. These results can be found on our website. Therefore the gene-finding accuracy for function-known genes of MED 2.0 and other five gene finders is well matched.

A distinctive feature of MED2.0 is that the algorithm builds a prokaryotic gene structure model based on a set of genome-specific parameters, which are calculated by several self-learning iterations without any prior knowledge or training data. Comparing the model parameters among different species, *e.g*. those of gene-related properties as TIS-upstream signals and usage of start codons ATG, GTG and TTG, would shed light on the study of genomic comparison and evolution for Bacteria and Archaea [18]. To this end, we present in Additional File 1 the parameters of start codon usage and motifs found upstream to TIS for each species, although a more detailed list is provided in the output file by our program for the user. Genomes in Additional File 1 are listed as two groups of Bacteria (the first 28 species) and Archaea (the last 12 species) and sorted alphabetically. Almost for all analysed species, ATG is the most common start codon (usage varying from 60% to 98%) compared with TTG and GTG. An exception is the *M. kandleri *genome, for which the three start codons are used nearly equally (showing 26% usage of ATG). In contrast, the usage of TTG to GTG is different for different species. For example, GTG in *C. glutamicum *is much more favored than TTG (23% *vs *6%); while TTG in *B. burgdorferi *is more favored than GTG (7% *vs *20%). For 12 archaeal genomes, the start codon usage predicted by MED 2.0 is very close to an earlier result annotated by EasyGene including the protein similarity search method [18].

With the automatic control coefficient *λ *in formula (6) and the resulting predicted motifs obtained by a non-supervised learning process, MED 2.0 clearly defines three types of translation initiation mechanisms and ascertains which of the types each genome belongs to. In Additional File 1, we go further and report the predicted motifs, meaning the most significant consensuses associated with translation initiation signals in two searching regions [-20, -1] and [-35, -15] upstream to TISs. For all of 28 analysed bacterial genomes, motifs are presented only in region [-20, -1], while most of them belong to SD signal type, which means the typical mechanism of translation initiation with the SD signals for transcripts with leaders. However, AT-rich motifs, "AATTT" and "AAATT" listed in Additional File 1, are detected in region [-20, -1] for the *Synechocystis sp*. genome, indicating the existence of a leadless translation initiation mechanism in Cyanobacteria. A similar case is to the *B. thetaiotaomicron *genome, where A-rich motifs are detected. For 12 analysed archaeal genomes, three types of mechanisms are detected. Six species *M. jannaschii, M. kandleri, M. maripaludis, M. thermoautotro., P. abyssi *and *P. furiosus *are reported with motifs of SD signal type only in region [-20, -1], meaning the mechanism similar to the bacterial. In contrast, three genomes *Halobacterium sp., N. equitans *and *P. aerophilum *are shown that the motifs of box A type are predicted only in region [-35, -15], which suggest a different mechanism of translation initiation with a high level of genes producing leaderless transcripts. For three genomes *A. fulgidus, P. torridus *and *S. solfataricus*, both box A motifs in region [-35, -15] for leaderless transcripts and SD motifs in [-20, -1] for transcripts with leaders are detected. It implies that a mixed mechanism of leaderless translation and leadered translation is used in these genomes. The result is well matched with that of previous studies [18,26].

A notable result is that most of the motifs of searching region [-20, -1] found in Bacteria contain the tetramer "GGAG" in contrast with that of "GGTG" in most Archaea, it is also exactly consistent with that reported in the earlier study [18]. However, note that for the *Thermotoga maritima *genome, the MED 2.0 analysis leads to an interesting finding of SD-like motifs very richly observed in Archaea. A phylogenetic tree based on 16S rRNA sequence has revealed that *T. maritima *is a deep branching species [36]. Moreover, it has been reported that over 20% of genes in *T. maritima *were acquired through horizontal gene transfer from Archaea [37]. Thus, the Archaea-like feature in *T. maritima *might be interpreted as either an ancient feature or, alternatively, a consequence of horizontal gene transfer.

### Accuracy of exact prediction on reliable genes

In order to perform an accuracy test on the exact gene prediction, reliable gene datasets must be used since the GenBank annotations have systematic bias in the 5' end prediction [15]. Before showing the results in this section, we should like to stress that all six gene finders, including MED 2.0, are compared to analyze each genome with the same sensitivity and specificity against the GenBank annotation as mentioned above. As a gene finder using a similarity search by alignment in Swiss-Prot to build TlS-confirmed ORFs, many a protein coding gene has been included as training set of TIS for EasyGene. For example, there are 679 of the 4,100 genes in *Bsub_All *dataset, and 356 of the 854 genes in *EcoGene*, have been employed as its training set for *B. subtilis *and *E. coli *respectively. Thus for a fair comparison with the *ab initio *methods, the accuracies of EasyGene on reliable test sets were calculated by excluding the number of genes included into its training set.

First we present the prediction accuracy on reliable test sets *Bsub_All, EcoGene *and *Link *for bacterial genomes. As indicated in Table 1, for only 3' end match, all six programs have nearly the same performance with accuracy above 98.0%. For both 5' and 3' end matches, the current version of GeneMarkS achieves the highest performance with average accuracy of 91.5%, while MED 2.0 has a comparative performance with average accuracy of 89.7%. Note that for *Bsub_All *dataset, GeneMarkS has reported its accuracy of 96.7% for 3' end match and 83.2% for both end matches on 4,099 genes annotated in GenBank [23], which is lower than the newest result (98.9% and 86.1%) by the current GeneMarkS version, as well as lower than that of MED 2.0 (98.7% and 83.8%) (see Table 1). ZCURVE 1.0 shows slightly lower average accuracy of 88.2% for both end matches, and EasyGene does 87.6%, while Glimmer 2.02 (post-processed by RBSfinder) gives the lowest average accuracy of 81.0%. Glimmer 3.02 has significantly improved the average accuracy to 89.6%, reaching a similar performance as MED 2.0.

One of the remaining challenges for prokaryotic gene finding is the identification of short genes 'buried' in an enormous pile of false-positive short ORFs. Following the standard adopted by many gene finders, we hereby define short genes as those with length between 90 and 300 bps. We have carried out a test on 58 short genes from 854 confirmed ones in *EcoGene *data set (denoted as *EcoGene_short*). The test indicates that MED 2.0 has an accuracy of 93.1% for 3' end match and also a high accuracy of 91.4% for both end matches. For the *Bsub123, Bsub72 *and *Bsub51 *datasets [23] from *B. subtilis*, MED 2.0 detects 95.1%, 94.4% and 92.2% of 3' end match and 85.4%, 87.5% and 90.2% of both end matches, respectively. Similarly, note that the corresponding published results of GeneMarkS on the same three datasets are 91.9%, 94.4% and 94.1% of 3' end match as well as 82.9%, 88.9% and 90.2% of both end matches [23], which have been greatly improved given by the newest online version. However as listed in Table 1, comparing with other five gene finders shows that MED 2.0 gives a higher performance in the short gene prediction than Glimmer 2.02 (post-processed by RBSfinder), Glimmer 3.02, EasyGene and ZCURVE 1.0, while is comparable with GeneMarkS.

**Table 1:** Prediction for 5' and 3' gene-ends for five programs on test sets. Comparison of prediction for 5' and 3' ends of genes are performed among MED 2.0 (MED), Glimmer 2.02 post-processed by RBSfinder (GL2), Glimmer 3.02 (GL3) GeneMarkS (GMK), ZCURVE 1.0 (ZCV) and EasyGene (EG) on a set of reliable test sets^*a*^

Test set^*b*^	Gene #	3' end match (%)						Both ends match (%)					
		MED	GL2	GL3	GMK^*c*^	ZCV	EG	MED	GL2	GL3	GMK^*c*^	ZCV	EG
Bsub_All	4100	98.7	98.2	97.6	98.9 (96.7)	98.4	94.5	83.8	75.0	82.4	86.1 (83.2)	83.1	79.6
EcoGene	854	99.1	99.3	99.4	99.9 (-)	98.8	99.4	92.0	82.5	91.9	93.8 (-)	89.2	91.1
Link	195	99.0	100.0	100.0	100.0 (100.0)	100.0	100.0	93.3	85.6	94.4	94.4 (94.4)	92.3	92.1
EcoGene_short	58	93.1	91.4	96.6	100.0 (-)	86.2	93.3	91.4	77.6	89.7	98.3 (-)	77.6	90.0
Bsub123	123	95.1	91.1	87.8	97.6 (91.9)	91.9	73.0	85.4	73.2	77.2	87.8 (82.9)	78.0	66.0
Bsub72	72	94.4	91.7	87.5	98.6 (94.4)	93.1	82.4	87.5	75.0	77.8	93.1 (88.9)	86.1	76.5
Bsub51	51	92.2	88.2	82.3	98.0 (94.1)	90.2	84.8	90.2	70.6	78.4	94.1(90.2)	84.3	81.8
Psaer107	107	97.2	100.0	95.3	93.5 (-)	95.3	100.0	93.5	83.2	90.6	85.0 (-)	91.6	88.0
Mtub66	66	95.5	98.5	97.0	98.5 (-)	97.0	97.5	87.9	60.6	80.3	80.3 (-)	75.8	82.5
SolfGene	56	100.0	100.0	100.0	100.0 (-)	100.0	100.0	89.3	50.0	87.5	85.7 (-)	73.2	89.3

^*a*^Programs MED, GL2 (post-processed by RBSfinder), GL3 and ZCV were run locally, while GMK was run online, as described in the text. Predictions for EG were downloaded from [34].

^*b*^Experiment confirmed TISs data sets: the first three represent two well-studied genomes: *B. subtilis *(Bsub_All) and *E. coli *(EcoGene and Link); the fourth to seventh represent short genes for *E. coli *(EcoGene_short) and *B. subtilis *(*Bsub123, Bsub72 *and *Bsub51*); Psaer107 and Mtub66 are selected for two GC rich genomes, *M. tuberculosis *(GC%: 65.6) and *P. aeruginosa *(GC%: 66.6); SolfGene corresponds to the archaeal *S. solfataricus*.

^*c*^Numbers in parentheses indicate that the results of GeneMarkS have been reported in literature, (-) means no data reported.

To evaluate the performance of exact gene prediction for GC-rich genomes, two reliable sets *Paer107 *and *Mtub66 *are used here for *P. aeruginosa *with GC content of 66.6% and *M. tuberculosis *with GC content of 65.6%. Test on *Paer107 *shows that MED 2.0 has an accuracy of 97.2% for 3' end match and 93.5% for both end matches, while on *Mtub66 *gives an accuracy of 95.5% for 3' end match and 87.9% for both end matches. As we can see from the eighth and ninth lines in Table 1, compared with Glimmer 2.02 (post-processed by RBSfinder), Glimmer 3.02, ZCURVE 1.0, EasyGene and the newest online version of GeneMarkS, MED 2.0 demonstrates the highest performance of exact gene prediction for GC-rich genomes. It is still problematic to evaluate gene prediction for archaeal genomes due to the insufficient number of genes confirmed by independent (*i.e*. non-computational) methods [18]. To this end, we have built the data set *SolfGene *comprising 56 reliable genes with confirmed TISs. All six programs detect all genes at the 3' end in *SolfGene*. As for both ends prediction, both MED 2.0 and EasyGene give the highest accuracy of 89.3% *vs *Glimmer 2.02 (post-processed by RBSfinder) (71.4%), Glimmer 3.02 (87.5%), GeneMarkS (85.7%), and ZCURVE 1.0 (73.2%) (See the last line in Table 1).

In summary, upon test of the reliable gene sets built for two well studied genomes *E. coli *and *B. subtilis*, we show that the total prediction performance of MED 2.0 can be matched with that of GeneMarkS in its publication, and higher than that of Glimmer, EasyGene and ZCURVE 1.0. While for the newly analysed genomes, especially for GC-rich genomes and Archaea, MED 2.0 shows an evident advantage, because test on the reliable gene sets clearly indicates that MED 2.0 has given a higher accuracy than other five gene finders, at least the same level of the best one.

### Analysis of upstream region of predicted TISs for Archaea

**Figure 2:**
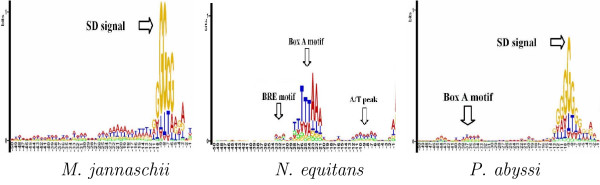
**Sequence logos of TIS-upstream-regions predicted by MED 2.0 for three archaeal genomes**. We present the logos of three representative archaeal genomes: *M. jannaschii, N. equitans *and *P. abyssi*. The logos of start codon at position 0 to +2 are masked off.

MED 2.0 gives a comprehensive modeling of the TIS for both bacterial and archaeal genomes, in particular for archeal genomes in which translation initiation is more complex than in Bacteria [18]. We report herein the results using the extended sequence logos [38] for the region [-50, -1] upstream from predicted TISs by MED 2.0. The sequence logos facilitate detection of sequence patterns that are conserved in both content and position. We present the logos of three representative archaeal genomes: *M. jannaschii, P. abyssi*, and *N. equitans *in Fig. 2. The logos indicate that several mechanisms associated with the translation initiation have been detected by MED 2.0. The genome of *M. jannaschii *displays the logos with a remarkable SD signals in region [-12, -4] like Bacteria, which is well compatible with the notion that this genome produces nearly no leaderless transcripts [18]. On the other hand, the *N. equitans *genome that carries a high level of genes producing leaderless transcripts [17,18] produces a very rich set of signals. An evident A/T-rich signal, the so-called box A promoter motif, is observed near the position -23. In addition, a TFB recognition element (BRE) motif constituting 2 to 4 A/Ts, which interact with the archaeal transcription factor TFB, is also recognized about 4 bps upstream from the box A motif. Furthermore, an A/T peak is detected near the position -9 to -10 upstream to the TIS, which has been reported being functionally important [18]. Finally, the *P. abyssi *genome is found to have a strongly conserved SD signal in -12 to -4 upstream region together with a rather weak box A motif upstream from the SD signal. This is well consistent with the fact that the *P. abyssi *genome produces fewer leaderless than leadered transcripts [17].

**Figure 3:**
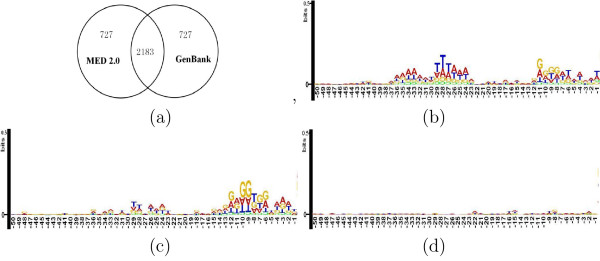
**Sequence logos of TIS-upstream-regions for MED prediction and GenBank annotation to *S. solfataricus***. (**a**) Venn diagram indicating the numbers of common and different gene starts given by MED 2.0 and GenBank; (**b**) Sequence logos of upstream region to TISs agreed by both MED 2.0 and GenBank; (**c**) Sequence logos of upstream region to TISs predicted only by MED 2.0; (**d**) Sequence logos of upstream region to TISs annotated only in GenBank. The logos of start codon at position 0 to +2 are masked off.

Inspection on the upstream sequence patterns of predicted TISs would be helpful for the assessment of the overall quality of gene annotation or prediction. Herein we choose an example, the *S. solfataricus *genome, for a detailed comparison between MED 2.0 and other prediction programs, as well as the GenBank annotation. First we contrast MED 2.0 with GenBank annotation. As we can calculate from Additional File 1, MED 2.0 detects 2910 genes with common 3' end against GenBank annotation, among which 2183 genes have common TISs and 727 genes do not (Fig. 3a). Fig. 3b shows the logos of the upstream region [-50, -1] from TISs of the common 2183 genes, where a typical SD signal can be observed at [-12, -4] and an evident box A motif at [-30, -23]. The logos for the other 727 TISs predicted by MED 2.0 are shown in Fig. 3c, where similar SD signal and box A motif are found, while those TISs from the GenBank annotation shown in Fig. 3d show no informational structure in the logos. This comparison suggests that MED 2.0 can give a more relevant annotation of TIS for archaeal genomes than the current GenBank annotation.

**Figure 4:**
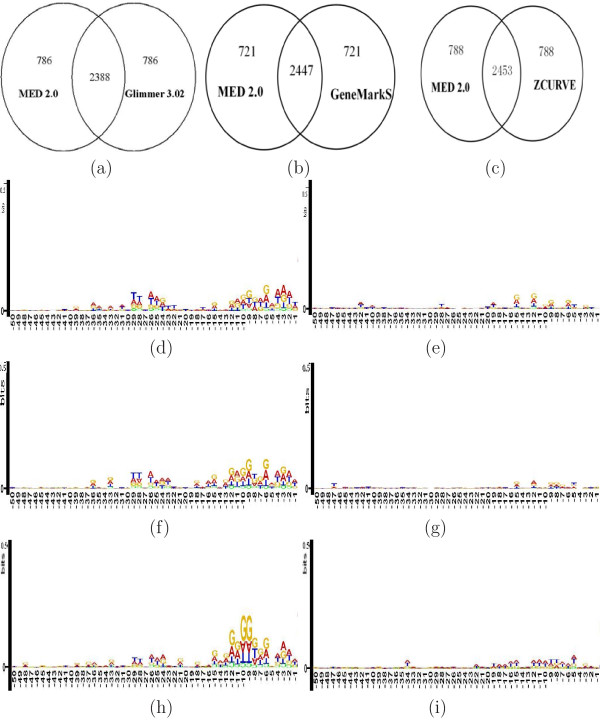
**Sequence logos of TIS-upstream-regions for MED, Glimmer, GeneMarkS and ZCURVE prediction to *S. solfataricus***. The three Venn diagrams indicate the number of common and different gene starts by MED 2.0 versus Glimmer 3.02 (**a**), GeneMarkS (**b**) and ZCURVE 1.0 (**c**), separately. The left side sequence logos are for upstream regions to the TISs predicted by MED2.0 but rejected by Glimmer 3.02 (**d**), GeneMarkS (**f**) and ZCURVE 1.0 (**h**). The right side sequence logos of are for upstream regions to the TISs predicted by Glimmer 3.02 (**e**), GeneMarkS (**g**) and ZCURVE 1.0 (**i**) but rejected by MED2.0. The logos of start codon at position 0 to +2 are masked off.

Similar comparison is made with Glimmer 3.02, GeneMarkS and ZCURVE 1.0, as shown in Fig. 4. We have shown that Glimmer 3.02 has achieved an overall improvement of TIS prediction than that of its earlier version, thus have no need for the comparison with Glimmer 2.02. The sets of common TIS predictions are slightly different in each case (see Fig. 4a,b and 4c), but in all comparisons the TISs predicted by MED 2.0 show the sequence logos similar to that in Fig. 3b (Fig. 4d,f and 4h), while those of Glimmer 3.02 (Fig. 4e), GeneMarkS (Fig. 4g) and of ZCURVE 1.0 (Fig. 4i) show almost no SD signal or box A motif structures. Thus to a great extent, MED 2.0 makes a substantially more accurate prediction than Glimmer 3.02, GeneMarkS and ZCURVE 1.0 for archaeal genomes.

## Conclusion

In this paper, we present a comprehensive model to describe a set of properties about the coding potential and the translation initiation mechanisms for both Bacteria and Archaea. Based on the model, with a design of multiple iterations, an non-supervised *ab initio *gene prediction system MED 2.0 is developed. Generally speaking, the system is able to adapt to any newly sequenced prokaryotic genome with no need for any data training or prior knowledge, and can predict the divergent translation initiation mechanisms and the resulting signals upstream from the TIS. The model seems to be biologically sound, since the program yields genome-specific model parameters such as various probabilities associated with the translation initiation signals and the start codon usage, which are matched with the current knowledge from earlier works. Thus the model may provide a good tool for comparative genomic studies. Upon test of a set of reliable gene sets, the total prediction performance of MED 2.0 for the well studied Bacteria such as *E. coli *and *B. subtilis *with usual GC content can be matched with that of the existing published methods. While for the newly analysed genomes, especially for GC-rich genomes and Archaea, test on the reliable gene sets indicates that MED 2.0 outperforms or at least gives the same level of the best of the current gene finders in exact gene prediction for both 3' and 5' end matches. Furthermore, MED 2.0 adapts to a broad range of archaeal genome as well as to bacterial genomes. For archaeal genomes with more complex mechanisms of translational initiation, our method has a more accurate prediction of TISs compared to the existing gene finders and the current GenBank annotation.

As many a prokaryotic gene prediction program, such as Glimmer, GeneMark and EasyGene, has been widely used in raw genomic sequence annotation, the predicting performance for a new gene prediction method evaluated by data extracted from GenBank should be in caution. While the comparison against the GenBank annotation has played a role during the early development of the computational prediction programs, this comparison becomes increasingly suspicious as its bias becomes evident (towards the programs which are used to create the GenBank annotation file). Therefore the consistency with the GenBank annotation should not be considered to be the only factor, and should not even be the essential one for some archaeal genomes. With hundreds of prokaryotic genomes have been sequenced and made publicly available, subsequent studies will focus more on the development of the comprehensive model of gene structure that allow capture of the biological evidence of prokaryotic genomes, *e.g*. the translation initiation mechanism, which is essential to understanding comparative genomics and biology evolution.

At present the problems of being biased and erroneous have not yet been solved in the GenBank annotation for prokaryotes, especially for newly sequenced archaeal genomes and GC-rich genomes, and the further promotion of comparative genomics, it is hoped that our system is shown to meet the demand and be qualified as an alternative tool of gene computational prediction. The expert annotators, who are concerned with more accurate and complete annotation of sequenced genomes, should find our method useful when used independently or alongside with other gene finding tools.

## Availability and requirements of the system

Project name: MED project;

Project homepage: ;

Operating systems: Microsoft Windows 2000 (or higher version) operating system is recommended for the pre-complied program downloaded from our web site. In addition, we also provide the source code on Linux with a C++ compiler;

Programming language: C++;

Licence: The sourc code is freely available (Additional File 2) under the GNU GPL license;

Any restrictions to use by non-academics: none.

## Authors' contributions

ZSS and HQZ co-supervised the whole development of the work and had the main idea of the algorithm, GQH and HQZ designed and implemented the gene-finder system, drafted most parts of the manuscript. YFY and JW contributed biological expertise and performed part of the experimental test. All authors read and approved the final manuscript.

## Supplementary Material

Additional file 1 **MED 2.0 prediction accuracy and genomic-specific model parameters**. contains data for MED prediction accuracy and genomic-specific model parameters for 28 bacterial genomes and 12 archaeal genomes. The file is edited by Microsoft Excel 2002. Click here for file

Additional file 2 **Source code**. contains source code of MED 2.0 written for WINDOWS and LINUX/UNIX operation systems. Click here for file
