# Supplementary material for: MED: a new non-supervised gene prediction algorithm for bacterial and archaeal genomes
Source: BMC Bioinformatics. 2007 Mar 16;8:97. doi: 10.1186/1471-2105-8-97 (PMC1847833; doi:10.1186/1471-2105-8-97)
Supplement: Additional file 2 — Source code. contains source code of MED 2.0 written for WINDOWS and LINUX/UNIX operation systems. [file 1471-2105-8-97-S2.zip › WINDOWS/MED2/MED2.plg]

```
# Build Log


### --------------------Configuration: MED2 - Win32 Release--------------------


### Command Lines

Creating temporary file "C:\DOCUME~1\fujitsu\LOCALS~1\Temp\RSP4F1.tmp" with contents
[
/nologo /ML /W3 /GX /O2 /D "WIN32" /D "NDEBUG" /D "_CONSOLE" /D "_MBCS" /Fp"Release/MED2.pch" /YX /Fo"Release/" /Fd"Release/" /FD /c 
"F:\PaperStudio\MED2_new\BMC_article2006_11_10\checkinglist\source code\WINDOWS\MED2\MED2\GeneInfo.cpp"
"F:\PaperStudio\MED2_new\BMC_article2006_11_10\checkinglist\source code\WINDOWS\MED2\MED2\GeneSeq.cpp"
"F:\PaperStudio\MED2_new\BMC_article2006_11_10\checkinglist\source code\WINDOWS\MED2\MED2\MainProcess.cpp"
"F:\PaperStudio\MED2_new\BMC_article2006_11_10\checkinglist\source code\WINDOWS\MED2\MED2\MED.CPP"
"F:\PaperStudio\MED2_new\BMC_article2006_11_10\checkinglist\source code\WINDOWS\MED2\MED2\OftenUsedOperatLib.cpp"
"F:\PaperStudio\MED2_new\BMC_article2006_11_10\checkinglist\source code\WINDOWS\MED2\MED2\SequenceTransform.cpp"
]
Creating command line "cl.exe @C:\DOCUME~1\fujitsu\LOCALS~1\Temp\RSP4F1.tmp" 
Creating temporary file "C:\DOCUME~1\fujitsu\LOCALS~1\Temp\RSP4F2.tmp" with contents
[
kernel32.lib user32.lib gdi32.lib winspool.lib comdlg32.lib advapi32.lib shell32.lib ole32.lib oleaut32.lib uuid.lib odbc32.lib odbccp32.lib kernel32.lib user32.lib gdi32.lib winspool.lib comdlg32.lib advapi32.lib shell32.lib ole32.lib oleaut32.lib uuid.lib odbc32.lib odbccp32.lib /nologo /subsystem:console /incremental:no /pdb:"Release/MED2.pdb" /machine:I386 /out:"Release/MED2.exe" 
".\Release\GeneInfo.obj"
".\Release\GeneSeq.obj"
".\Release\MainProcess.obj"
".\Release\MED.OBJ"
".\Release\MED_start.obj"
".\Release\OftenUsedOperatLib.obj"
".\Release\SequenceTransform.obj"
]
Creating command line "link.exe @C:\DOCUME~1\fujitsu\LOCALS~1\Temp\RSP4F2.tmp"

### Output Window

Compiling...
GeneInfo.cpp
GeneSeq.cpp
MainProcess.cpp
MED.CPP
OftenUsedOperatLib.cpp
SequenceTransform.cpp
Linking...

### Results

MED2.exe - 0 error(s), 0 warning(s)
```
