# Supplementary material for: MED: a new non-supervised gene prediction algorithm for bacterial and archaeal genomes
Source: BMC Bioinformatics. 2007 Mar 16;8:97. doi: 10.1186/1471-2105-8-97 (PMC1847833; doi:10.1186/1471-2105-8-97)
Supplement: Additional file 2 — Source code. contains source code of MED 2.0 written for WINDOWS and LINUX/UNIX operation systems. [file 1471-2105-8-97-S2.zip › WINDOWS/TISModel/TISModel.plg]

```
# Build Log


### --------------------Configuration: TISModel - Win32 Release--------------------


### Command Lines


### Results

TISModel.exe - 0 error(s), 0 warning(s)
```
